# Video-based interviewing in medicine: a scoping review

**DOI:** 10.1186/s13643-022-01959-8

**Published:** 2022-05-16

**Authors:** Rajajee Selvam, Richard Hu, Reilly Musselman, Isabelle Raiche, Daniel I. McIsaac, Husein Moloo

**Affiliations:** 1 Department of Surgery, The Ottawa Hospital, Ottawa, Ontario K1Y 1J8 Canada; 2 The Ottawa Hospital Research Institute, Ottawa, Ontario Canada; 3 Departments of Anesthesiology & Pain Medicine, The Ottawa Hospital, University of Ottawa, Ottawa, Canada; 4 School of Epidemiology & Public Health, University of Ottawa, Ottawa, Canada

**Keywords:** Video-based interviews, Videoconferencing, Personnel selection, Climate change

## Abstract

**Background:**

The Coronavirus 2019 pandemic necessitated a rapid uptake of video-based interviewing within the personnel selection process in healthcare. While video-based interviews have been evaluated previously, we identified a gap in the literature on the implementation of video-based interviews and how they compare to their face-to-face counterparts.

**Methods:**

A scoping review was conducted to consolidate the available literature on the benefits and limitations of video-based interviews and to understand the perceived barriers associated with transitioning away from face-to-face interviews. A search strategy, developed in concert with an academic health sciences librarian, was run on Ovid MEDLINE, Embase, PsycInfo, and Cochrane Central. The search was performed on March 31, 2020, and updated on February 21, 2021. Studies that implemented and evaluated the impact of video-based interviewing in healthcare were included in our study. Review articles and editorials were excluded.

**Results:**

Forty-three studies were included in our scoping review, of which 17 were conference abstracts and 26 were peer-reviewed manuscripts. The risk of bias was moderate or high in most studies, with only four studies having a low risk of bias. Both financial costs and opportunity costs associated with the selection process were reported to be improved with video-based interviewing, while no studies explored the impact on environmental costs. Technical limitations, which were not prevalent, were easily managed during the interview process. Overall, video-based interviews were well received by both applicants and interviewers, although most participants still reported a preference for face-to-face interviews.

**Conclusions:**

While video-based interviewing has become necessary during the Coronavirus 2019 era, there are benefits from a financial, opportunistic, and environmental point of view that argue for its continued use even after the pandemic. Despite its successful implementation with minimal technical issues, a preference still remains for face-to-face interviews. Reasons for this preference are not clear from the available literature. Future studies on the role of nonverbal communication during the video-based interview process are important to better understand how video-based interviewing can be optimized.

**Systematic review registration:**

This scoping review was registered with Open Science Framework.

**Supplementary Information:**

The online version contains supplementary material available at 10.1186/s13643-022-01959-8.

## Background

Whether applying to medical school, residency programs, fellowships, or for the role of a staff physician, interviews play an important part of the selection process for both applicants and programs. The Coronavirus 2019 (COVID-19) pandemic, and the public health measures that have accompanied it, has necessitated a transition away from face-to-face interviews. This provides an opportunity to scrutinize the benefits and limitations of video-based interviewing as they compare to traditional face-to-face interviews.

Face-to-face interviews come with both financial and opportunity costs that have been reported in previous studies [1–4]. Financial costs result from application fees, travel and accommodation for interviews, and costs associated with completing elective rotations [1–3]. Opportunity costs such as time taken away from clinical service to attend interviews, which applies to applicants and interviewer, should also be considered [3, 4].

The environmental costs of face-to-face interviews have not been well-studied in the literature, but a recent emphasis has been placed on the impact of long-haul flights associated with traveling for interviews on our carbon footprint [5]. In line with this, the healthcare sector is regarded as a major contributor to greenhouse gas emissions, urging for a call to action towards climate change as a public health emergency [6, 7].

Video-based interviewing presents an opportunity to continue with personnel selection by adhering to COVID-19 restrictions while addressing the aforementioned costs associated with face-to-face interviews. While previous studies have reported on their experience with video-based interviews and provided summaries of the current literature on the topic, a formal structured synthesis of the literature on video-based interviewing within healthcare does not exist [8–13]. Given the rapid transition to video-based interviewing, an opportunity was identified to consolidate the current literature on the topic.

## Methods

### Study aim

The aim of this review was to evaluate the extent of previous research on video-based interviewing as it applies to healthcare contexts. We sought to review the benefits and limitations of video-based interviewing and identify gaps in our current knowledge on the implementation of video-based interviewing. The research question that guided this scoping review was as follows:

*Within selection processes in healthcare, how do video-based interviews compare with face-to-face interviews in terms of costs, implementation, and candidate selection?*

### Study design

A scoping review was conducted in anticipation of the heterogeneity of study designs available on this topic. We employed the methodology that has been previously outlined and subsequently refined [14, 15]. Full details to the conduct of this scoping review can be found in our previously published study protocol [16]. In short, a search strategy developed in conjunction with an academic health sciences librarian was used with the following databases: Ovid MEDLINE, Embase, PsycInfo, and Cochrane Central. The search was run on March 31, 2020, to capture studies from inception to March 30, 2020. The gray literature was searched using Google, and the reference list of all studies selected for inclusion was reviewed for any additional studies that fit our inclusion criteria. Our study was reported according to the Preferred Reporting Items for Systematic reviews and Meta-Analyses extension for Scoping Reviews (PRISMA-ScR) checklist, which is provided in Additional file 1 [17]. The search strategy can be found in Additional file 2. The search was updated on February 20, 2021.

Inclusion and exclusion criteria that were developed to select studies that were in line with our research question are provided below. Studies that were not published in English or French were excluded due to translation services available. There was no limitation based on publication date.

Inclusion criteria:

- Involve applicants interviewing via video-based and/or face-to-face interviews AND
- Involve applicants applying to medical school, residency, fellowship programs, dentistry, pharmacy, nursing, or other healthcare-related fields AND
- Any study design that involved the implementation of video-based interviews AND
- Any method of data analysis, including quantitative and qualitative studies AND
- Assess any outcome of interest including financial costs, environmental impact, or time invested

Exclusion criteria:

- Review articles OR
- Editorials or expert opinions that do not describe a particular video-based interview that was implemented OR
- Studies that are not published in English or French

### Data acquisition

The results of our search were initially screened based on title and abstract by two independent reviewers (RS, RH). Cohen’s kappa statistic was determined following this to ensure inter-rater reliability before proceeding to the next stage of screening. Any discrepant studies by either of these reviewers were included in full text review. After initial screen based on title and abstract, the two reviewers (RS, RH) independently screened the full texts of the selected studies in duplicate. Any discrepancies following this were reviewed by a third independent reviewer (HM) who made the final decision on study inclusion. Reasons for exclusion after full text review were documented.

DistillerSR (Evidence Partners, Ottawa, Canada) was used for the data extraction process. A standardized form was created on this platform and independently tested by two reviewers (RS and RH) on the initial 10 studies that were included. Following this, the data extraction form was modified to capture the range of methodologies and outcomes of the studies that were included in our scoping review. No further changes were made to the data extraction form after this modification, and the same standardized form was used on all included studies.

### Data analysis

Given the nature of a scoping review, no formal statistical synthesis of the study data was pursued. Study characteristics including geographic location, study methodology, discipline of authors, purpose of interview, and type of analysis are presented as descriptive data. In line with our aim to identify the benefits and limitations of video-based interviewing, outcomes from each study were also grouped thematically as they applied to financial costs, opportunity costs, environmental costs, technical limitations, impact on the rank list, and body language.

### Quality assessment

Risk of bias assessment of each included article was performed using the Joanna Briggs Institute (JBI) critical appraisal tools by two independent reviewers (RS, RH) [18]. Although not a typical component of a scoping review, we sought to provide an assessment of the study quality available in the current literature. Abstracts were not critically appraised as they lacked the methodological detail required to assess for quality and risk of bias. The tools relevant for cohort studies, cross-sectional studies, randomized controlled trials, and quasi-experimental studies were used as applicable. Based on the results of the JBI critical appraisal tool, an overall study rating was assigned by the reviewers to describe the risk of bias as either “high,” “moderate,” or “low” risk. All studies that met the inclusion criteria were included in our scoping review, regardless of the results of their quality assessment.

## Results

### Search results

**Fig. 1:**
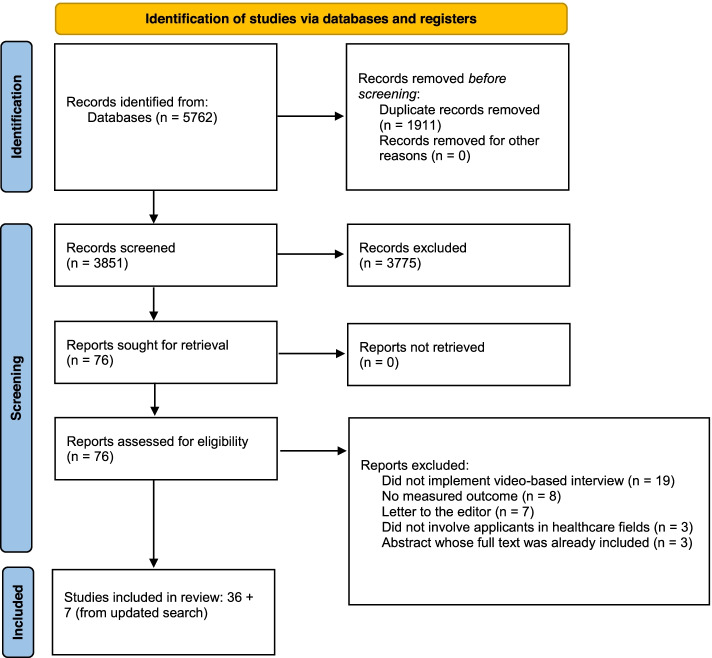
PRISMA 2020 flow diagram for new systematic reviews which included searches of databases and registers only. *From: *Page MJ, McKenzie JE, Bossuyt PM, Boutron I, Hoffmann TC, Mulrow CD, et al. The PRISMA 2020 statement: an updated guideline for reporting systematic reviews. BMJ 2021;372:n71. doi: 10.1136/bmj.n71. For more information, visit: http://www.prisma-statement.org/

A total of 3851 studies were retrieved from our search, after duplicates were removed. Following review of titles and abstracts by two independent reviewers, a total of 76 studies were selected for full text review (Cohen’s kappa = 0.84). Following independent full text review by two reviewers, a total of 37 studies were selected. These were reviewed by our third reviewer after which 36 articles were included. Reasons for exclusion after full text review included the lack of any video-based interview being implemented (*n* = 19), letters to the editor (*n* = 7), did not involve applicants in healthcare fields (*n* = 3), did not have any measured outcomes (*n* = 8), or abstracts which full text was already included (*n* = 3). The search was updated on February 20, 2021, and 5 additional studies met inclusion criteria. Two additional studies were retrieved from review of the references of the included studies. As such, a total of 43 studies were included in our scoping review [19–61]. A PRISMA flow diagram is provided in Fig. 1 to outline the study selection process [62]. A summary of the demographic data and eligibility criteria of the included studies is provided in Table 1. Our outcomes, which were group thematically, are summarized separately in Table 2.

**Table 1:** Characteristics of included studies

Author	Year published	Title	City, country	Discipline of authors	Format of study	Sample size		Purpose of interview	Study design	Match cycle	Intervention	Comparator	Replacement for face-to-face interview or adjunct?
						Number of interviewers	Number of applicants						
Winfield-Dial et al.	2018	Demographic differences between high and low scorers on the standardized video interview	Chicago, USA	Emergency medicine	Abstract	None	1440	Medical school students applying to residency	Cross-sectional study	2018	Standardized video interview (SVI)	None	Adjunct
Winfield-Dial et al.	2018	Applicant attitudes towards the standardized video interview—an interim analysis	Chicago, USA	Emergency medicine	Abstract	None	80	Medical school students applying to residency	Cross-sectional study	2018	Standardized video interview (SVI)	None	Adjunct
Humbert et al.	2018	Correlation of the standard video interview score with an established application review process	Indiana, USA	Emergency medicine	Abstract	None	964	Medical school students applying to residency	Cross-sectional study	2018	Standardized video interview (SVI)	None	Adjunct
Naemi et al.	2019	Examining the relationship between the AAMC standardized video interview and step 2 CS subscores	Washington, USA	Emergency medicine	Abstract	None	2201	Medical school students applying to residency	Cross-sectional study	2017	Standardized video interview (SVI)	None	Adjunct
Chukwumah et al.	2010	The use of remote computer audio-video processing to conduct surgical fellowship interviews of deployed physicians	Cleveland, USA	General surgery	Abstract	None	26	Residents applying to fellowships	Cross-sectional study	2011	Skype panel interview	None	Replacement
Chandler et al.	2019	Efficacy of videoconference interviews in the pediatric surgery match	Florida, USA	Pediatric surgery	Journal article	3	20	Residents applying to fellowships	Cross-sectional study	2017	Videoconference interview	Initial virtual interview followed by face-to-face interview	Adjunct
Chung et al.	2019	How well does the standardized video interview score correlate with traditional interview performance?	New York, USA	Emergency medicine	Journal article	None	321	Medical school students applying to residency	Cross-sectional study	2018	Standardized video interview (SVI)	Face-to-face panel interview	Adjunct
Brietkpof et al.	2018	One-way video interviewing as a method to augment the residency application	Minnesota, USA	Obstetrics and gynecology and orthopedic surgery	Abstract	None	57	Medical school students applying to residency	Cross-sectional study	2017	One-way video interview	Face-to-face panel interview	Adjunct
Tiller at al.	2013	Internet-based multiple mini-interviews for candidate selection for graduate entry programs	Sydney, Australia	Faculty of medicine	Journal article	78	999	Students applying to medical or dental school	Cohort study	2009 - 2011	Skype multiple mini-interviews	Face-to-face multiple mini-interview	Replacement
Brietkpof et al.	2019	Use of asynchronous video interviews for selecting obstetrics and gynecology residents.	Minnesota, USA	Obstetrics/gynecology	Journal article	None	219	Medical school students applying to residency	Cross-sectional study	2018 - 2019	Asynchronous video interview	Face-to-face panel interview	Adjunct
Daram et al.	2014	Interview from anywhere: feasibility and utility of web-based videoconference interviews in the gastroenterology fellowship selection process	Mississippi, USA	Gastroenterology	Journal article	None	16	Residents applying to fellowships	Cross-sectional study	2013	Facetime panel interview	Face-to-face interview	Adjunct
Deiorio et al.	2019	Applicant reactions to the AAMC standardized video interview during the 2018 application cycle	United States	Emergency medicine	Journal article	None	3532	Medical school students applying to residency	Cross-sectional study	2018	Standardized Video Interview (SVI)	None	Adjunct
Hakes et al.	2018	Communication and professionalism: comparing standardized video interview scores to faculty gestalt	Wisconsin, USA	Emergency medicine	Abstract	None	65	Medical school students applying to residency	Cross-sectional study	2018	Standardized Video Interview (SVI)	None	Adjunct
Edje et al.	2013	Using Skype as an alternative for residency selection interviews	Ohio, USA	Family medicine	Journal article	11	19	Medical school students applying to residency	Cohort study	2012	Skype panel interview	Face-to-face panel interview	Adjunct
Egan et al.	2019	Standardized video interviews do not correlate to US medical licensing examination step 1 and step 2 scores	New York, USA	Emergency medicine	Journal article	None	1329	Medical school students applying to residency	Cross-sectional study	2018	Standardized video interview (SVI)	None	Adjunct
Gallahue at al.	2019	The AAMC standardized video interview: reactions and use by residency programs during the 2018 application cycle	USA	Emergency medicine	Journal article	125	3532	Medical school students applying to residency	Cross-sectional study	2018	Standardized video interview (SVI)	None	Adjunct
Healy et al.	2017	Videoconference interviews for an adult reconstruction fellowship: lessons learned	Massachusetts, USA	Orthopedic surgery	Journal article	Not reported	47	Residents applying to fellowships	Cross-sectional study	2015 - 2017	Skype panel interview	None	Replacement
Hopson et al.	2019	Comparison of the standardized video interview and interview assessments of professionalism and interpersonal communication skills in emergency medicine	USA	Emergency medicine	Journal article	151	773	Medical school students applying to residency	Cross-sectional study	2018	Standardized video interview (SVI)	None	Adjunct
Hopson et al.	2019	The AAMC standardized video interview and the electronic standardized letter of evaluation in emergency medicine: a comparison of performance characteristics	USA	Emergency medicine	Journal article	None	2884	Medical school students applying to residency	Cross-sectional study	2018	Standardized video interview (SVI)	None	Adjunct
Husain et al.	2019	The standardized video interview: how does it affect the likelihood to invite for a residency interview?	USA	Emergency medicine	Journal article	None	1424	Medical school students applying to residency	Cross-sectional study	2018	Standardized video interview (SVI)	None	Adjunct
Lewis et al.	2018	Standardized video interview scores do not correlate with attending evaluations	Massachusetts, USA	Emergency medicine	Abstract	None	24	Medical school students applying to residency	Cross-sectional study	2018	Standardized video interview (SVI)	None	Adjunct
Willis et al.	2018	Are standardized video interview scores predictive of interview performance?	New York, USA	Emergency medicine	Abstract	None	57	Medical school students applying to residency	Cross--sectional study	2018	Standardized video interview (SVI)	None	Adjunct
Bowers et al.	2019	Standard video interview scores and applicant position on residency program list: a correlation study	Ohio, USA	Emergency medicine	Abstract	None	1003	Medical school students applying to residency	Cross-sectional study	2018	Standardized video interview (SVI)	None	Adjunct
Hall et al.	2018	Standard video interview score does not correlate with medical student communication skills	Massachusetts, USA	Emergency medicine	Abstract	None	19	Medical school students applying to residency	Cross-sectional study	2018	Standardized video interview (SVI)	None	Adjunct
McHugh et al.	2019	Do standardized or traditional interview questions correlate with the standardized video interview?	USA	Emergency medicine	Abstract	None	98	Medical school students applying to residency	Cohort study	2018	Standardized video interview (SVI)	None	Adjunct
Staicu et al.	2015	FaceTime face-off: evaluation of video conferencing as a novel pre-interview screen for a PGY-1 pharmacy residency	New York, USA	Pharmacy	Abstract	None	23	Pharmacy students applying to pharmacy residency	Cross-sectional study	2015	Skype/FaceTime panel interview	None	Adjunct
Temple et al.	2014	Streamlining the residency interview process using web-based teleconferencing	Cleveland, USA	Pharmacy	Journal article	None	24	Pharmacy students applying to pharmacy residency	Cross-sectional study	2013	Skype panel interview	None	Adjunct
Hall et al.	2019	Standardized video interview scores correlate poorly with faculty and patient ratings	Massachusetts, USA	Emergency medicine	Journal article	58	36	Medical school students applying to residency	Cross-sectional study	2018	Standardized video interview (SVI)	None	Adjunct
Ballejos et al.	2018	An equivalence study of interview platform: does videoconference technology impact medical school acceptance rates of different groups?	New Mexico, USA	Family medicine/emergency medicine	Journal article	None	752	Students applying to medical school	Quasi-experimental study	2014 - 2016	Skype panel interview	Face-to-face panel interview	Replacement
Bird et al.	2019	Innovation in residency selection: the AAMC standardized video interview	USA	Emergency medicine	Journal article	None	4387	Medical school students applying to residency	Cross-sectional study	2017 - 2018	Standardized video interview (SVI)	None	Adjunct
Schnapp et al.	2019	Assessing residency applicants’ communication and professionalism: standardized video interview scores compared to faculty gestalt	Wisconsin, USA	Emergency medicine	Journal article	None	125	Medical school students applying to residency	Cross-sectional study	2018	Standardized video interview (SVI)	None	Adjunct
Shah et al.	2018	The standardized video interview: how well does the SVI score correlate with traditional interview performance?	UA	Emergency medicine	Abstract	None	97	Medical school students applying to residency	Cross-sectional study	2018	Standardized video interview (SVI)	Face-to-face panel interview	Adjunct
Shah et al.	2012	Randomized evaluation of a web-based interview process for urology resident selection	New Mexico, USA	Urology	Journal article	6	33	Medical school students applying to residency	Randomized trial	2011	Skype panel interview	Face-to-face panel interview	Replacement
Vadi et al.	2016	Comparison of web-based and face-to-face interviews for application to an anesthesiology training program: a pilot study	California	Anesthesia	Journal article	None	169	Medical school students applying to residency	Quasi-experimental study	2015	Skype/FaceTime panel interview	Face-to-face panel interview	Replacement
Krauss et al.	2018	Correlation between emergency medicine residency applicant’s standardized video interview scores and US medical licensing examination results	USA	Emergency medicine	Abstract	None	1329	Medical school students applying to residency	Cross-sectional study	2018	Standardized video interview (SVI)	None	Adjunct
Williams et al.	2015	Videoconference interviewing: tips for success	Arizona, USA	Internal medicine	Journal article	None	6	Medical school students applying to residency	Cross-sectional study	2014	Skype panel interview	None	Replacement
Molina et al.	2020	Virtual interviews for the complex general surgical oncology fellowship: the Dana-Farber/Partners Experience	Boston, USA	Complex general surgical oncology	Journal article	Not reported	Not reported	Residents applying to fellowships	Cross-sectional study	2020	Zoom panel interview	Face-to-face panel interview	Replacement
Sripad	2020	Videoconference interviews for female pelvic medicine and reconstructive surgery fellowship during a pandemic: the candidate experience	Rhode Island, USA	Female pelvic medicine and reconstructive surgery	Abstract	None	14	Residents applying to fellowships	Cross-sectional study	2020	Zoom panel interview	None	Replacement
Nutter et al.	2020	Perception of candidates and faculty on maternal fetal medicine fellowship videoconference interviewing	Texas, USA	Maternal fetal medicine	Abstract	Not reported	14	Residents applying to fellowships	Cross-sectional study	2018-2019	Videoconference panel interview	None	Replacement
McAteer et al.	2020	Videoconference interviews: a timely primary care residency selection approach	New York, USA	Family medicine	Journal article	Not reported	39	Medical school students applying to residency	Cross-sectional study	2011-2020	Skype panel interview	None	Adjunct
Majumder et al.	2020	Initial experience with a virtual platform for advanced gastrointestinal minimally invasive surgery fellowship interviews	Missouri, USA	Advanced gastrointestinal minimally invasive surgery	Journal article	7	17	Residents applying to fellowships	Cross-sectional study	2019-2020	Zoom panel interview	None	Replacement
Grova et al.	2020	Direct comparison of in-person versus virtual interviews for complex general surgical oncology fellowship in the COVID-19 era	North Carolina, USA	Complex general surgical oncology	Journal article	None	23	Residents applying to fellowships	Cohort study	2020	Zoom panel interview	Face-to-face panel interview	Replacement
Vining et al.	2020	Virtual surgical fellowship recruitment during COVID-19 and its implications for resident/fellow recruitment in the future	Illinois, USA	Complex general surgical oncology	Journal article	12	16	Residents applying to fellowships	Cross-sectional study	2020	Zoom panel interview	None	Replacement

Author	Interview information				Methods		
	Description	Platform	Pre-interview information	Number of interviewers	Primary outcomes	Secondary outcomes	Type of analysis
Winfield-Dial et al.	SVI			N/A	Demographic (sex, race/ethnicity, medical school type, age, and step 1 score) differences between those that scored high vs. low on the SVI	None	Quantitative: chi-squared test
Winfield-Dial et al.	SVI			N/A	Survey responses on applicants’ thoughts on the added value of the SVI	None	Quantitative: descriptive
Humbert et al.	SVI			N/A	Correlation between internally developed composite score and SVI score	Correlation between internally developed composite score and SVI score with whether an interview was offered	Quantitative: Pearson correlation, point-biserial correlations
Naemi et al.	SVI			N/A	Correlation between SVI score and step 2 CS exam subscores (CIS, SEP, ICE)	None	Quantitative: Pearson correlation
Chukwumah et al.	One videoconference interview (no further details provided)	Skype	None	Not reported	None	None	None
Chandler et al.	Three 20-min interviews; applicants were ranked by each faculty before and after the virtual interview, and all applicants were invited for a face-to-face interview	Skype	Prior to interview, applicants were sent a detailed information packet outlining the fellowship program, instructions on how to set up a Skype account, and were asked to create their accounts 2 weeks prior	3 different faculty members	15 question survey for applicants and 8 question survey for faculty to assess perceptions regarding ease and convenience of virtual interview, ability to represent oneself, decision if the program and/or applicant is the right fit, and utility as a screening tool and/or substitute for on-site interview	Movement on pre-virtual interview rank list to post-virtual interview rank list; cost to applicants from interview process	Quantitative: descriptive, Student’s *t*-test, Fisher’s exact test
Chung et al.	SVI			N/A	Correlation between SVI score and traditional interview score	Variance of traditional interview score with SVI subgroup scores (6–11, 12–17, 18–23, 24–30)	Quantitative: linear regression, ANOVA
Brietkpof et al.	One-way video interview with 3 questions and max. 3 min per questions	Not reported	None	N/A (videos were reviewed and scored independently by 2 reviewers using a standardized 5-point scale)	Correlation between one-way video interview scores and in-person interview scores	Applicant satisfaction with one-way video interview	Quantitative: correlation, descriptive
Tiller at al.	7-min questions with 2-min change over time; candidates were on their own laptop; interviewers rotated through 9 computer stations in a large room; 5 administrative staff and two IT staff; candidate reads the first line of scenario out loud to confirm they received the correct prompt	Skype	All applicants started with a meeting with IT 30 min prior to ensure good connectivity	One interviewer per station	Equivalence between in-person and online MMI (based on applicant scores)	Reliability, feasibility, acceptability, and cost-effectiveness of virtual MMI	Quantitative: descriptive, ANOVA, Levene’s statistic
Brietkpof et al.	Three questions that were developed after a pilot with medical students; applicants were able to view each question for 2 min prior to starting their recording of their answers (3-min response time per questions); applicants were allowed to re-record their answers once if desired	Montage Talent	None	N/A	Does video interviewing improve the overall pool of candidates as measured by higher in-person interview scores?	Applicant experience captured by survey	Quantitative: descriptive; two-tailed *t*-test, chi-squared test, Pearson correlation, Spearman rank correlation
Daram et al.	One video interview	Facetime	None	1	Survey responses on whether the virtual interview met their expectations	Costs associated with interview process	Quantitative: descriptive
Deiorio et al.	SVI			N/A	Survey responses on applicants’ preparation for the SVI and reactions to the procedural aspects of SVI	Survey responses on applicants’ perceptions of the SVI experience and future of the selection process	Quantitative: descriptive
Hakes et al.	SVI			N/A	Correlation between SVI scores and faculty gestalt scores	None	Quantitative: Pearson correlation
Edje et al.	Three sequential, 25-min interviews	Skype	None	2	Cost savings with Skype interview	Survey responses on applicants’ and interviewers’ perspectives on Skype interview	Quantitative: descriptive
Egan et al.	SVI			N/A	Correlation between SVI and step 1 and step 2 CK scores	Correlation between SVI and step 2 CS scores	Quantitative: linear regression, Kruskal-Wallis test, Mann-Whitney *U*-test
Gallahue at al.	SVI			N/A	Survey responses of program directors' perceptions on SVI	Number of views of each SVI by the program	Quantitative: descriptive, Cohen’s *h*-test, *t*-tests
Healy et al.	One 15–20 min interview	Skype	Video tour of facility	2–3	Survey responses of applicants’ experience with videoconference interview	Faculty opinions	Quantitative: descriptive
Hopson et al.	SVI			N/A	Correlation between SVI and interviewer-scored professionalism and interpersonal communication skills	Correlation of SVI and rank list; influence of gender on assessment of professionalism/interpersonal communication skills	Quantitative: ANOVA, *t*-tests
Hopson et al.	SVI			N/A	Correlation between electronic standardized letter of evaluation (eSLOE) and SVI	Correlation between eSLOE/SVI on rotation grades, USMLE scores, honor society memberships	Quantitative: Spearman rank correlations, point-biserial correlations, Pearson correlations, Cohen’s d
Husain et al.	SVI			N/A	Likelihood to invite for interview (LTI) pre-SVI score reviewed, post-SVI score reviewed, and post-SVI video viewed	Subgroup analysis by USMLE score and SVI score	Quantitative: *t*-test, ANOVA, linear regression
Lewis et al.	SVI			N/A	Correlation between SVI scores and attending evaluations of professionalism and patient care/communication performance during EM clerkship	None	Quantitative: Spearman rank correlations
Willis et al.	SVI			N/A	Correlation between SVI score and interview score	None	Quantitative: Spearman rank correlations
Bowers et al.	SVI			N/A	Correlation between SVI score and position on rank list	None	Quantitative: correlation
Hall et al.	SVI			N/A	Correlation between SVI and patient assessment of communication (communication assessment tool)	None	Quantitative: Spearman’s rank correlation
McHugh et al.	SVI			N/A	Correlation between traditional interview score, standardized interview score, and SVI score	None	Quantitative: descriptive, ANOVA
Staicu et al.	One 15-min interview with five behavioral-based questions	Skype or FaceTime	None	3 (residency director, coordinator, and a preceptor)	Technical issues, number invited for on-site interview	None	Quantitative: descriptive
Temple et al.	One 20-minu interview; 5 behavioral-based questions; candidates had 5 min to ask questions; total possible score of 30	Skype or FaceTime	None	2 (clinical pharmacy specialists or administrator)	Description of interview conduct, financial costs, and time spent	None	Quantitative: descriptive
Hall et al.	SVI			N/A	Correlation between SVI and faculty evaluations on professionalism and patient care/communication	Correlation between SVI and patient reported communication skills (CAT)	Quantitative: Spearman’s rank correlation
Ballejos et al.	One-on-one semi-structured 30–60 min interview	Skype	None	1	Medical school acceptance rate using videoconference vs. face-to-face interview	Impact of socioeconomic status, self-identified disadvantaged category on acceptance rate	Quantitative: descriptive, *t*-test, chi-squared test
Bird et al.	SVI			N/A	Demographic differences in SVI score	Correlations between SVI scores and other measures (USMLE step scores, honor society memberships, etc.)	Quantitative: descriptive, rater reliability, *t*-test, Pearson correlation, point-biserial correlations, Cohen’s d
Schnapp et al.	SVI			N/A	Correlation between SVI and faculty gestalt score	Correlation between overall interview score and SVI	Quantitative: Spearman’s rank correlation
Shah et al.	SVI			N/A	Correlation between SVI and in-person interview scores	None	Quantitative: inear regression
Shah et al.	One-on-one 15-min interviews	Skype	Video tour of facilities; opportunity to ask residents questions; brief Skype test call to coordinator 1 week before interview	3–6 different faculty	Survey responses of applicant/faculty perspectives on effectiveness of web-based interview	Comparison of rank list position between web-based versus on-site interview; financial cost; educational cost	Quantitative: descriptive, Mann-Whitney *U*-test, Fisher’s exact test
Vadi et al.	Three/four 10-min interviews with faculty	FaceTime or Skype	Audio/video version of program overview; video tour of medical center and surrounding communities; google hangout session with current residents; option to schedule an on-campus department tour	6 faculty (number of interviews per interview not clear)	Proportion of applicants selected via face-to-face vs. web-based interview	Survey responses of applicants’ perspectives of web-based interview	Quantitative: Shapiro-Wilk test, *t*-test, Wilcoxon rank-sum test, chi-square test, Wald test
Krauss et al.	SVI			N/A	Correlation between USMLE scores and SVI scores	None	Quantitative: linear regression, Kruskal-Wallis test
Williams et al.	One 30-min interview	Skype	Virtual tour with commentary by chief residents; electronic brochures; resident contact info provided	4 (program director, associate program director, and 2 chief residents)	Survey responses from applicants about their experience	None	Quantitative: descriptive
Molina et al.	Five 15-min interviews using break out rooms on Zoom. Program coordinator moved applicants/faculty between breakout rooms on Zoom	Zoom	30-min general overview provided by program director; semi-structured fellow’s “room” where current fellows showed a pre-recorded virtual tour of the hospitals, presentation on research opportunities, and topics of interest to prospective fellows	2–4 faculty interviewers per interview “room”	Survey responses from applicants on conduct of the virtual interview compared to those from previous year on conduct of face-to-face interview	Survey responses from faculty on conduct of virtual interview	Quantitative: descriptive
Sripad	One 30-min interview	Zoom	Option to meet current fellows during an informal videoconference the night prior to their interview; applicants were sent a 15-min information video about the program; 15-min introductory presentation by program director on interview day	2–4 faculty/fellows	Survey responses from applicants on their experience	None	Quantitative: descriptive
Nutter et al.	One panel interview	Not reported	Prior to interviews, candidates were provided with a link to a PowerPoint presentation and virtual campus tour; candidates were offered contact information for additional questions and to visit campus at their leisure	Five interviewers	Survey responses of applicants	Survey responses of interviewers	Quantitative: descriptive, student *t*-test, Mann-Whitney *U*-test
McAteer et al.	One-on-one panel interview	Skype	None	1	Cost and time savings with virtual interview	Survey responses of applicants and faculty	Quantitative: descriptive
Majumder et al.	One-on-one panel interviews	Zoom	Presentation overview of the program; orientation to Zoom and the use of breakout rooms; informal breakout room with current fellows	5–7 different faculty	Survey responses of applicants’ perspectives on the feasibility, appropriateness, and acceptability of virtual interview process	Survey responses of faculty's perspective of virtual interview process	Quantitative: descriptive
Grova et al.	Twelve one-on-one panel interviews	Zoom	Videoconference information session by program director; breakout room for informal interactions with fellows/faculty	12 different faculty members	Survey responses of applicants’ perspective of the interview experience	None	Quantitative: descriptive, *t*-tests
Vining et al.	Five panel interviews: 10 min with program director, 15 min with institutional leader, three 30-min interviews with faculty	Zoom	Optional session for applicants to get an overview of the program from program director and to meet staff members who would be points of contact for technical difficulties	Program director, institutional leads and three different faculties (total of 13 faculty members participated)	Survey responses from applicants	Survey responses from faculty	Quantitative: descriptive

**Table 2:** Study outcomes grouped thematically

Author	Year published	Title	Environmental costs	Financial costs	Opportunity costs	Technological issues	Body Language	Impact on Rank List	Study Limitations
Winfield-Dial et al.	2018	Demographic differences between high and low scorers on the standardized video interview	Not reported	Not reported	Not reported	Not reported	Not reported	Not reported	Not reported
Winfield-Dial et al.	2018	Applicant attitudes towards the standardized video interview—an interim analysis	Not reported	Not reported	Not reported	Not reported	Not reported	Not reported	Not reported
Humbert et al.	2018	Correlation of the standard video interview score with an established application review process	Not reported	Not reported	Not reported	Not reported	Not reported	Not reported	Not reported
Naemi et al.	2019	Examining the relationship between the AAMC standardized video interview and step 2 CS subscores	Not reported	Not reported	Not reported	Not reported	Not reported	Not reported	Not reported
Chukwumah et al.	2010	The use of remote computer audio-video processing to conduct surgical fellowship interviews of deployed physicians	Not reported	Not reported	Not reported	Not reported	Not reported	Not reported	Not reported
Chandler et al.	2019	Efficacy of videoconference interviews in the pediatric surgery match	Not reported	Financial cost was a hardship for applicants; video conferencing comes without the cost and inconvenience of travel	90% of applicants reported that the amount of time spent for interviews was a hardship; applicants may appear more fatigued or stressed when interviewing during or after a work day	Occasional trouble connecting when the applicants were interviewing from a hospital	Not reported	Some applicants moved up on rank list following videoconference interview	Recall bias; reliability of survey data; applicants may be biased to provide positive responses (controlled for by administering survey after submission of rank lists)
Chung et al.	2019	How well does the standardized video interview score correlate with traditional interview performance?	Not reported	Not reported	Not reported	Not reported	Not reported	Not reported	Sample size; traditional interviews were not structured (low interrater reliability amongst interviewers)
Brietkpof et al.	2018	One-way video interviewing as a method to augment the residency application	Not reported	Not reported	Not reported	Not reported	Not reported	Positive correlation between one-way interview score and rank list position	Not reported
Tiller at al.	2013	Internet-based multiple mini-interviews for candidate selection for graduate entry programs	Not reported	$50000/year of cost savings for university; substantial cost savings for applicants	Virtual interview provided time savings for applicants	No significant technical concerns, need for some improvement in audio/visual quality	Not reported	Not reported	Naturalistic study design (can’t control for crossover); low survey response rate
Brietkpof et al.	2019	Use of asynchronous video interviews for selecting obstetrics and gynecology residents	Not reported	Not reported	Delayed in-person interviews by 3 weeks, but this did not have a significant effect on the number of applicants accepting in-person interviews; increased burden of work for program	Not reported	Not reported	Trend towards positive correlation between asynchronous interview sore and rank list position	Not reported
Daram et al.	2014	Interview from anywhere: feasibility and utility of web-based videoconference interviews in the gastroenterology fellowship selection process	Not reported	Web-based videoconference interviewing provided cost savings for applicants	Avoiding interview date conflicts; avoiding need to take time off from work	Logistics are simple (high-speed Internet, smart phones)	Not reported	Not reported	Small sample size, lack of randomization, selection bias
Deiorio et al.	2019	Applicant reactions to the AAMC standardized video interview during the 2018 application cycle	Not reported	Not reported	Not reported	Not reported	Not reported	Not reported	Recall bias; limited length of surveys
Hakes et al.	2018	Communication and professionalism: comparing standardized video interview scores to faculty gestalt	Not reported	Not reported	Not reported	Not reported	Not reported	Not reported	Not reported
Edje et al.	2013	Using Skype as an alternative for residency selection interviews	Not reported	There are cost savings for both applicants and interviewers	There are time savings for applicants and interviewers	Voice delay with Skype interviews	Lack of physical contact such as a hand shake was more of a concern for interviewers	Not reported	Not reported
Egan et al.	2019	Standardized video interviews do not correlate to US medical licensing examination step 1 and step 2 scores	Not reported	Not reported	Not reported	Not reported	Not reported	Not reported	Not all applicants from 2017 to 2017 application season were included which may limit generalizability
Gallahue at al.	2019	The AAMC standardized video interview: reactions and use by residency programs during the 2018 application cycle	Not reported	Not reported	Not reported	Not reported	Not reported	SVI scores may not be useful in determining who to invite for an interview	Skewed responses to survey questions; hard to expand on their reactions with surveys (qualitative study may be useful); video usage did not reflect the duration of how long the SVI was viewed for
Healy et al.	2017	Videoconference interviews for an adult reconstruction fellowship: lessons learned	Not reported	Not reported	Reduced time spent on interview for applicants and faculty	Not reported	Not reported	Most applicants were comfortable ranking a program after a videoconference interview	Not reported
Hopson et al.	2019	Comparison of the standardized video interview and interview assessments of professionalism and interpersonal communication skills in emergency medicine	Not reported	Not reported	Not reported	Not reported	Not reported	Not reported	No standardized interview protocol across programs; no valid scoring system available for professionalism or interpersonal/communication skills
Hopson et al.	2019	The AAMC standardized video interview and the electronic standardized letter of evaluation in emergency medicine: a comparison of performance characteristics	Not reported	Not reported	Not reported	Not reported	Not reported	Not reported	Did not assess the practical significance of SVI/eSLOE correlations
Husain et al.	2019	The standardized video interview: how does it affect the likelihood to invite for a residency interview	Not reported	Not reported	Not reported	Not reported	Not reported	SVI score changed the likelihood to invite for an interview in 7% of applicants	No standardization of the selection process; faculty reviewers were NOT blinded to the study purpose; limited experience with SVI to begin with; no standardization to how long the reviewers should watch the videos
Lewis et al.	2018	Standardized video interview scores do not correlate with attending evaluations	Not reported	Not reported	Not reported	Not reported	Not reported	Not reported	Not reported
Willis et al.	2018	Are standardized video interview scores predictive of interview performance?	Not reported	Not reported	Not reported	Not reported	Not reported	Not reported	Small sample size
Bowers et al.	2019	Standard video interview scores and applicant position on residency program list: a correlation study	Not reported	Not reported	Not reported	Not reported	Not reported	No correlation between SVI scores and rank list	Not reported
Hall et al.	2018	Standard video interview score does not correlate with medical student communication skills	Not reported	Not reported	Not reported	Not reported	Not reported	Not reported	Not reported
McHugh et al.	2019	Do standardized or traditional interview questions correlate with the standardized video interview?	Not reported	Not reported	Not reported	Not reported	Not reported	Not reported	Not reported
Staicu et al.	2015	FaceTime face-off: evaluation of video conferencing as a novel pre-interview screen for a PGY-1 pharmacy residency	Not reported	Video conference interviews reduce applicant travel expenses	Minimal time investment required for videoconference interviews	Unsuccessful video in 22% of interviews, replaced with telephone interviews	Not reported	Not reported	Not reported
Temple et al.	2014	Streamlining the residency interview process using web-based teleconferencing	Not reported	Monetary savings from decreased cost for meals for onsite interviews	Less time taken away from patient care/clinical activity	Few interviews conducted via telephone due to connectivity issues	Loss of video connection prevented evaluation of body language which was part of the evaluation	Not reported	No evaluation of how applicants felt about video interviews; did not confirm identity of applicant
Hall et al.	2019	Standardized video interview scores correlate poorly with faculty and patient ratings	Not reported	Not reported	Not reported	Not reported	Not reported	Not reported	Small sample size; faculty evaluations group patient care and communication together; inter-rater reliability of faculty evaluations is questionable
Ballejos et al.	2018	An equivalence study of interview platform: does videoconference technology impact medical school acceptance rates of different groups?	Not reported	Not reported	Not reported	Not reported	Not reported	No significant change in acceptance rate between face-to-face vs. video interview	Conducted at single medical school; small sample size interviewed by video; may not be generalizable to other schools that are less diverse/rural
Bird et al.	2019	Innovation in residency selection: the AAMC standardized video interview	Not reported	Not reported	Unintended consequence of SVI may be increase in time required for preparation	Not reported	Not reported	Not reported	Other aspects of selection process like LOE, trainee performance outcomes not assessed; unclear if use of non-physician raters reduced the accuracy of SVI scores
Schnapp et al.	2019	Assessing residency applicants' communication and professionalism: standardized video interview scores compared to faculty gestalt	Not reported	SVI may not remain free	Not reported	Not reported	Not reported	Not reported	Small sample size; difficult to differentiate between faculty ratings of 1–25; no formalized protocol on how professionalism/communication should be assessed
Shah et al.	2018	The standardized video interview: how well does the SVI score correlate with traditional interview performance?	Not reported	Not reported	Not reported	Not reported	Not reported	Not reported	Not reported
Shah et al.	2012	Randomized evaluation of a web-based interview process for urology resident selection	Not reported	Cost savings for both applicants and programs	Less time taken away from school for applicants	Poor connection quality in several instances; had to re-connect via skype	Not reported	Similar distribution of applicants from each interview type on rank list	Average travel costs may be unestimated given geographic distribution of applicants; change in rank list position during study period may be secondary to improvement in applicant credentials
Vadi et al.	2016	Comparison of web-based and face-to-face interviews for application to an anesthesiology training program: a pilot study	Not reported	Video interviews selected due to financial costs in 25%	Video interviews selected due to inability to get time off in 9.4%	6.3% and 3.1% reported sub-optimal video and audio quality, respectively	Not reported	Interview type did not have a significant impact on rank list	No randomization to interview type; single-center study; single specialty
Krauss et al.	2018	Correlation between emergency medicine residency applicant’s standardized video interview scores and US medical licensing examination results	Not reported	Not reported	Not reported	Not reported	Not reported	Not reported	Not reported
Williams et al.	2015	Videoconference interviewing: tips for success	Not reported	Not reported	Not reported	Poor video quality reported 17% of applicants	Not reported	Most applicants felt that videoconference interview was sufficient for them to determine their rank list	Virtual interview was limited to 30 min, which may have affected program’s ability to convey aspects of the program
Molina et al.	2020	Virtual interviews for the complex general surgical oncology fellowship: the Dana-Farber/Partners Experience	Not reported	Virtual interview experience eliminated the burden of associated costs for the program itself with regard to food and drink but also the cost of travel for applicants	Decreased inconvenience of travel for applicants with virtual interviews; able to maximize the number of faculty members participating in the selection process with virtual interviews	Not reported	Not reported	Not reported	Not reported
Sripad	2020	Videoconference interviews for female pelvic medicine and reconstructive surgery fellowship during a pandemic: the candidate experience	Not reported	Not reported	Not reported	Not reported	Not reported	Not reported	Not reported
Nutter et al.	2020	Perception of candidates and faculty on maternal fetal medicine fellowship videoconference interviewing	Not reported	Benefit of videoconference interview is cost savings	Benefit of videoconference interview is time savings	Not reported	Not reported	Lack of subjective details from personal interaction	Not reported
McAteer et al.	2020	Videoconference interviews: a timely primary care residency selection approach	Not reported	55% reduction in costs to the program with initial videoconference interview to screen applicants	Not reported	Not reported	Not reported	Not reported	Cost estimate does not account for benefits of potential income generated through increased faculty and resident clinical productivity (owing to fewer in-person interview days); unable to quantify benefit of flexible scheduling with virtual interviews; variation in interview process over the years; poor response rates and non-standardized survey questions
Majumder et al.	2020	Initial experience with a virtual platform for advanced gastrointestinal minimally invasive surgery fellowship interviews	Not reported	89% of applicants reported cost savings as a strength of virtual interviewing	45% reported a reduction in missed time and improvement in flexibility of scheduling as a benefit of virtual interviewing	33% of applicants mentioned technical issues	Not reported	Not reported	No comparative control in-person interview group; small sample size; bias for applicants to provide favorable responses
Grova et al.	2020	Direct comparison of in-person versus virtual interviews for complex general surgical oncology fellowship in the COVID-19 era	Not reported	Not reported	Not reported	Not reported	Only 54% of applicants in the virtual interview group, compared to 92% from the face-to-face group, felt that the interview experience was sufficient to make a ranking decision	Virtual interviews need to improve the applicant’s ability to gain a feel of the culture of a program and to make a ranking decision	Single-institution study with limited sample size; recall bias; applicants may be biased toward more favorable responses as survey was administered prior to submission of rank lists
Vining et al.	2020	Virtual surgical fellowship recruitment during COVID-19 and its implications for resident/fellow recruitment in the future	Not reported	Applicants highlighted cost savings	Applicants highlight time savings	Faculty expressed ongoing nervousness about technical issues; only one faculty had a temporary technical connectivity problem	Not reported	Not reported	Not reported

### Study characteristics

Of the included studies, 17 were conference abstracts and 26 were published manuscripts. Forty-two studies were conducted in the USA, while 1 was conducted in Australia.

Twenty-nine studies included medical students applying to residency, 2 studies of students applying to medical/dental school, 2 studies of pharmacy students applying to their respective pharmacy residency, and 10 studies of residents applying to fellowship. Of studies involving medical residency or fellowship applications, disciplines included urology (*n* = 1), anesthesiology (*n* = 1), general surgery (*n* = 1), pediatric surgery (*n* = 1), gastroenterology (*n* = 1), internal medicine (*n* = 1), orthopedic surgery (*n* = 2), obstetrics and gynecology (*n* = 2), family medicine (*n* = 2), complex general surgical oncology (*n* = 3), female pelvic medicine and reconstructive surgery (*n* = 1), maternal fetal medicine (*n* = 1), advanced gastrointestinal minimally invasive surgery (*n* = 1), and emergency medicine (*n* = 22).

There was a range of study methodology. There were 36 cross-sectional studies, 4 cohort studies, 2 quasi-experimental studies, and 1 randomized trial. All studies employed some form of quantitative analysis, while one abstract did not outline what analysis they performed. No studies used qualitative methods.

### Virtual panel interview

Eighteen studies reported on their use of panel style virtual interviews. Of these, 6 were adjuncts [19–24] to the face-to-face interview while 12 were replacements [25–36]. Studies used Skype [19, 21, 24–28, 30], FaceTime [19], Zoom [31, 32, 34–36], or a combination of two platforms [22, 23, 29]. Eleven studies provided additional information outside the virtual interview itself including a video tour of the facility, video tour of the surrounding communities, Google hangout session with current residents, electronic brochures, and resident contact information to ask additional questions if interested [19, 26, 28–36]. The number of interviews varied from a single 15–30 min video-based interview [20, 22–27, 30, 32, 33] to multiple interviews with different faculty members [19, 21, 28, 29, 31, 34–36]. The number of interviewers also varied from one-on-one interviews [19, 20, 24, 27, 28, 34, 35] to those with up to five interviewers [22, 23, 26, 30–33]. Three studies described having a dedicated individual (e.g., program coordinator, information technology specialist) who facilitated movement of applicants and interviewers between breakout rooms on the video-based platform [31, 34, 35]. Three studies had an administrative staff responsible for ensuring that applicants had established appropriate audio/visual connections [19, 27, 28].

Nine studies reported favorable perceptions from applicants on video-based panel interviews [20, 21, 24, 26, 31, 32, 34–36]. Applicants in these studies felt that they were able to demonstrate their strengths and personality through a video-based format [26, 32, 35, 36], that it was a fair way to present the program to them [21], and that the interviews flowed well [31]. Most applicants found that the video-based interviews met their expectations [20, 26], and they were satisfied with the process [34].

In one study, applicants felt that the video-based interview was less effective in allowing them to represent themselves [28]. An additional study highlighted that video-based interviewing prevented the “gut feelings” about a program that are typically felt at the time of a face-to-face interview [33]. Most applicants in one study also found that the inability to see the city or meet the faculty face-to-face was a drawback to video-based interviews [34]. Other concerns with video-based interviewing included not being able to understand the program’s culture [35] and interact with current residents [29].

Few studies explored the interviewers’ perspective on video-based interviewing and found that interviewers were mostly satisfied with the video-based interview process overall [26, 31, 34]. In one study however, none of the interviewers recommended using video-based interviews as the sole method of interviewing [21].

In the 12 studies that specifically assessed the role of video-based interviewing as a replacement to the current process, two found that it worked well to replace face-to-face interview [31, 34], while 10 studies felt that it was a useful adjunct or screening tool, but was not ready to replace face-to-face interviews entirely [19–22, 26, 28, 32, 33, 35, 36].

Overall, the panel-based video interview format appeared to be acceptable to applicants and interviewers, although a complete transition away from face-to-face interviews has been met with hesitancy.

### Virtual multiple mini-interviews

One study reported on their use of video-based multiple mini-interviews (MMIs) [37]. With their retrospective cohort study comparing applicants to medical and dental school between years that employed face-to-face MMIs with those that employed video-based MMIs, they found that there was no significant difference between interview scores, although there was greater variability in scores in the video-based MMI group.

A total of 76% of applicants and 78% of interviewers were satisfied with the video-based MMI process. Similar to the face-to-face counterpart, the video-based MMIs involved seven questions with 2-min change over time between stations. The interview process was set up and overseen by five administrative staff and two IT staff. This study was the only study that explored the role of a video-based MMI as a replacement to the face-to-face counterpart. This one study demonstrated that the video-based MMI was an acceptable alternative to an in-person process.

### One-way video interviewing

One-way video interviewing describes the process by which applicants submit answers to standardized questions in video format to be evaluated as part of the selection process. There were 24 studies that employed this method of video-based interviewing.

Twenty-two of these studies evaluated the standardized video interview (SVI), an online unidirectional interview that was developed by the Association of American Medical Colleges (AAMC) and piloted with the Accreditation Council for Graduate Medical Education (ACGME)-accredited emergency medicine programs. Twelve of these studies were abstracts [38–49] and 10 were articles [50–59]. Applicants submitted an audio/video response to six questions which was subsequently scored from 6 to 30. The goal of the SVI was to provide standardized information about applicant’s interpersonal and communication skills and professionalism. It was introduced in 2016 as a research project and was administered as an operational pilot in the emergency medicine residency selection during the 2018, 2019, and 2020 match cycles. Although the SVI was not continued for the 2021 match cycle, these studies were included in our scoping review to assess the value of unidirectional interviewing.

Overall, there were small correlations between SVI scores and other aspects of the application such as USMLE scores [41], faculty scores of communication and professionalism [51], electronic standardized letter of evaluation (eLOE) [55], patient evaluation of communication skills [46], and traditional interview scores [47, 48]. One study questioned the utility of the SVI as there was a lack of a relationship between SVI scores and applicant ranking [40], while another study found that the SVI score changed the likelihood of a program to invite the applicant for an interview in 7% of cases [56]. Of studies that explored the applicant perspective, two found that applicants did not feel that the SVI should be part of the application process [39, 51].

Apart from the studies that assessed the AAMC’s SVI specifically, two other studies described their use of one-way video interviewing or asynchronous video interviews [60, 61]. Both these studies utilized one-way interviewing as an adjunct to face-to-face interviews, rather than a replacement. Applicants submitted a video response to three standardized questions that were scored to determine which applicants would be subsequently invited for a face-to-face interview.

Higher scores on this one-way interview were correlated with higher in-person interview scores [60, 61]. While one study reported a positive correlation between one-way interview score and rank list placement [60], the other study reported a nonsignificant positive correlation between the two [61].

Overall, results were ambiguous as to what the SVI was measuring and the value that should be attributed to it amongst other aspects of the emergency medicine selection process. Nonetheless, one-way interviews may hold some promise as an initial screen, but there is no evidence we found to demonstrate that it should replace a bidirectional interview.

### Technical limitations

Eleven of the included studies discussed technical limitations of video-based interviewing [19–23, 28–30, 34, 36, 37]. Three studies discussed issues with connectivity that were resolved by reconnecting or switching to telephone interviews without video [22, 23, 28]. One study discussed the voice delay associated with using the Skype platform for video-based interviews [21]. One study explained that while faculty was nervous about potential technical issues with video-based interviews, only one interviewer had a temporary technical connectivity problem [36].

No studies reported any major concerns from a technical point of view that limited the use of video-based interviewing.

### Financial cost

Fourteen studies discussed the financial costs associated with the selection proces s[19–24, 28, 29, 31, 33, 34, 36, 37, 59]. Thirteen of these studies reported a reduction in financial costs for either applicants [19, 20, 22, 33, 34, 36], programs/interviewers [23, 24, 29], or both [21, 28, 31, 37]. One study reported that applicants who matched successfully spent significantly more money compared to those that did not match [19]. In another study where applicants were given the option of interview format, 25% of applicants chose a video-based interview due to financial limitations [29]. Additionally, the importance of modifying the surgical fellowship recruitment process given increases in student debt had been alluded to by one study [36].

Overall, video-based interviewing was seen as a way to improve financial costs for all stakeholders, particularly for applicants in whom financial limitations impact their application process.

### Opportunity costs

Fifteen studies discussed the opportunity costs associated with the selection process [19–23, 26, 27, 29, 31, 33, 34, 36, 37, 58, 61]. Eleven of these studies reported that video-based interviewing afforded the applicants the ability to expend less time with the interview process [19–23, 26, 28, 33, 34, 36, 37], which would mean less time being taken away from clinical or educational commitments [20, 21, 28, 34].

Two studies discussed that video-based interviewing was particularly beneficial for applicants that could not get time off work to attend face-to-face interviews [29] and for applicants who would not be able to attend the face-to-face interview due to interview scheduling conflicts [20]. One study also highlighted that residency programs themselves would experience fewer disruptions with video-based interviewing since residents could take less time away from clinical duties [36].

One study explained that the introduction of the SVI may have increased the time required for applicants to prepare for the interview itself [28]. From a program’s perspective, one study reported that the utilization of one-way video interviewing prior to face-to-face interviews delayed the face-to-face interviews by 3 weeks and increased the burden of work from the program [61].

While one-way video interviewing may come at the cost of increased work for both applicants and interviewers, bidirectional video-based interviewing allows applicants to take less time away from personal or professional commitments and results in fewer disruptions for the programs themselves.

### Environmental cost

None of the 43 studies discussed the environmental impact of interviewing in healthcare as it applies to personnel selection.

### Body language

Three studies discussed the role of body language as it pertained to video-based interviewing [21, 23, 33]. One study commented on the lack of physical contact such as a hand shake but explained that this was more of a concern for interviewers [21]. Another study discussed how the loss of video connection in some interviews prevented the assessment of body language which was part of the evaluation [23]. Finally, one study commented on the lack of subjective details from an interaction that are lost in a video-based format [33].

None of the studies discussed the interpretation of body language and how this plays into applicant selection.

### Influence on rank list

There were 12 studies that assessed either the perceived or objective impact of video-based interviews on the rank list [19, 26–30, 35, 45, 56, 53, 60, 61]. Two of these studies reported a positive relationship between video-based interview scores and overall rank list [60, 61]. Three studies found that there were no differences in acceptance rate and/or rank list position based on whether applicants had a face-to-face or video-based interview [27–29].

Three studies reported from the applicants’ perspective that most applicants were comfortable ranking programs after a video-based interview [26, 30]. One study reported that only 54% of applicants who had a virtual interview felt that the experience was sufficient to make a ranking decision, compared to 92% of their counterparts who had a face-to-face interview experience [35].

From the interviewer perspective of determining the rank list, three studies reported that there was no significant difference of interview type on the ranking of applicants [28, 29].

In terms of the SVI, one study reported the lack of any significant correlation between the SVI scores and rank list position [45], while another study reported that the SVI score changed the likelihood of inviting an applicant for a face-to-face interview in 7% of cases, with lower SVI scores more likely to decrease the chance of an interview invite than higher scores were to increase the chance of an interview invite [56].

### Quality assessment

Quality assessment was completed for the 26 peer-reviewed manuscripts included in our scoping review. Using the JBI critical appraisal tools to evaluate each article, 11 studies were felt to have a high risk of bias, 12 with a moderate risk of bias, and only 3 studies with a low risk of bias. Most studies lacked a valid and reliable tool to evaluate applicant/interviewer perspectives on video-based interviews, did not assess or control for potential confounders, or had poor survey response rates. A summary of our quality assessment is provided in Additional file 3.

## Discussion

Our scoping review summarizes the current literature and highlights major themes about video-based interviewing in healthcare. Overall, both financial costs and opportunity costs associated with the selection process were reported to be improved with video-based interviewing, while the impact on environmental costs has not been well explored in the current literature. Bidirectional video-based interviews were well received by both applicants and interviewers, yet a preference remains for face-to-face interviews. One-way video interviewing may be useful in select settings as a screening tool, but was not found to be a good replacement for bidirectional interviewing.

Important features of video-based interviewing that were described by multiple studies included the use of a video tour of the facility/city, an informal video-based session with current trainees, and having a dedicated administrative person to help applicants and interviews navigate through the video-based platform. While technical limitations were anticipated, there were no major technical issues that limited the use of video-based interviews. The interpretation of body language in a video-based setting was not well explored.

In the context of the social distancing measures necessitated by the COVID-19 pandemic, video-based interviewing has garnered recent interest across multiple disciplines within healthcare which were captured by our broad search strategy. Our search was also updated after its initial run to capture the influx of studies that were published since the start of the COVID-19 pandemic. Previous reviews have not been as structured or encompassing of video-based interviewing in healthcare as our scoping review [13, 63], and it has been reported that developments addressing medical school/residency interviews are underreported [63].

The results of our review are in line with those from a previous literature review but also serve to highlight gaps in literature. Joshi and colleagues outlined benefits and drawbacks to video-based interviewing [13]. Similar benefits were found in our study including the decrease in financial costs and time taken away from clinical or academic duties. While Joshi and colleagues allude to the reduction in carbon footprint with video-based interviews, they also did not find any studies that evaluated this aspect.

An important finding in our review was that despite studies reporting that applicants and/or interviewers felt that the video-based interview worked well, completing transitioning away from face-to-face interviews was not seen as a favorable option. From the results of our review, however, reasons for this are unclear. One of the challenges with assessing the reasons for this hesitancy may be the methodology of the available studies. While using surveys to quantify applicant and interviewer perspectives on video-based interviewing is helpful, it falls short in highlighting the thought processes that guide decision-making. It has been suggested that qualitative studies that use an inductive approach to data gathering may be more beneficial in such circumstances to get a better understanding of the reasoning behind our decision-making [64].

Another aspect of video-based interviewing that was not well reported in the included studies is the role of body language and nonverbal communication. While the lack of body language was alluded to in three studies, quantifying the impact of body language on the video-based selection process has inherent challenges and has not been assessed in a healthcare context. In a study by Proost and colleagues from the organizational psychology literature, it was found that applicants are less attracted to organizations that use video-based interviews compared to those that use face-to-face interviews for their selection process [65]. They explain that this perception may be influenced by the decreased ability to convey and interpret nonverbal cues on a virtual platform. While it has been studied on the organizational psychology literature, the results of our scoping review suggest that nonverbal communication as it applies to video-based interviewing in medicine is an area for future research.

Given the nature of the topic, the challenges associated with survey studies, and the inherent biases involved in the selection process, most studies had a moderate or high risk of bias. Additionally, analyzing perceptions around video-based interviewing comes with the challenge of measuring differing viewpoints in a reliable and valid way. We did not come across any validated measurement tool as studies developed and implemented their own surveys. While the goal of our scoping review was not to statistically synthesize data, we appreciate that collating the results of these heterogenous studies has its difficulties. Future research on developing an assessment tool to grade the quality of the interviews may prove helpful in standardizing the evaluation of interview processes.

## Conclusion

Our scoping review summarizes the available literature on the use of video-based interviewing in healthcare contexts and has highlighted important areas for further exploration. Video-based interviewing, while necessary during the COVID-19 era, provides benefits from a financial, opportunistic, and environmental point of view that argue for its continued use even after the pandemic. While video-based interviewing has been reported to be a feasible alternative to face-to-face interviewing, hesitancy remains to completely transition from face-to-face interview for reasons that are difficult to extrapolate from the currently available literature. In addition, the role of nonverbal communication and how this factors into decision-making is important to evaluate, as it will help to better understand the complex thought processes that underlie personnel selection in healthcare.

## Supplementary Information

**Additional file 1:.** Preferred Reporting Items for Systematic reviews and Meta-Analyses extension for Scoping Reviews (PRISMA-ScR) Checklist

**Additional file 2:.** Database: Ovid MEDLINE(R) ALL <1946 to February 19, 2021>. Search Strategy

**Additional file 3:.** Table 2a: Risk of Bias Assessment for Cohort Studies. Table 2b: Risk of Bias Assessment for Randomized Trials. Table 2c: Risk of Bias Assessment for Quasi Experimental Studies. Table 2d: Risk of Bias Assessment for Cross Sectional Studies

## Data Availability

To ensure transparency and reproducibility, all data generated or analyzed during this study has been included in the published scoping review article and/or its supplementary information files. This includes the search strategy, reasons for study exclusion, and extracted data used in analysis.

## Abbreviations

COVID-19: Coronavirus disease 2019
PRISMA: Preferred Reporting Items for Systematic Reviews and Meta-analyses
PRISMA-ScR: PRISMA extension for Scoping Reviews
JBI: Joanna Briggs Institute
SVI: Standardized video interview
AAMC: Association of American Medical Colleges
ACGME: Accreditation Council for Graduate Medical Education
eLOE: Electronic standardized letter of evaluation
