# Supplementary material for: Video-based interviewing in medicine: a scoping review
Source: Syst Rev. 2022 May 16;11:94. doi: 10.1186/s13643-022-01959-8 (PMC9108136; doi:10.1186/s13643-022-01959-8)
Supplement: Supplementary file 2 — Additional file 2:. Database: Ovid MEDLINE(R) ALL <1946 to February 19, 2021>. Search Strategy [file 13643_2022_1959_MOESM2_ESM.docx]

Database: Ovid MEDLINE(R) ALL <1946 to February 19, 2021>

Search Strategy:

--------------------------------------------------------------------------------

1 Interviews as topic/ or Interview.ti,ab. (194399)

2 Personnel Selection/ or personnel selection.ti,ab. (13153)

3 1 or 2 (206221)

4 Videoconferencing/ (1791)

5 ((video* or skype* or whatsapp* or facetime* or web* or internet* or tele* or remote*) adj3 interview*).ti,ab. (22038)

6 4 or 5 (23776)

7 3 and 6 (12074)

8 exp education, medical/ or education, medical, continuing/ or education, medical, graduate/ or "internship and residency"/ or education, medical, undergraduate/ (169100)

9 exp Specialties, Surgical/ed [Education] (29151)

10 (medical student* or residency or medical residency or internship* or residency application* or fellowship* or fellow*).ti,ab. (97600)

11 8 or 9 or 10 (235914)

12 Climate change/ (18363)

13 Carbon footprint/ (657)

14 "Costs and Cost Analysis"/ (49311)

15 environmental pollution/ or petroleum pollution/ or traffic-related pollution/ (21045)

16 (environment* or climate* or carbon footprint or financial* or cost*).ti,ab. (1783797)

17 12 or 13 or 14 or 15 or 16 (1816819)

18 11 or 17 (2032826)

19 7 and 18 (1938)

20 7 and 18 (1938)

21 limit 20 to yr="2020 - 2021" (138)
