# Supplementary material for: Video-based interviewing in medicine: a scoping review
Source: Syst Rev. 2022 May 16;11:94. doi: 10.1186/s13643-022-01959-8 (PMC9108136; doi:10.1186/s13643-022-01959-8)
Supplement: Supplementary file 3 — Additional file 3:. Table 2a: Risk of Bias Assessment for Cohort Studies. Table 2b: Risk of Bias Assessment for Randomized Trials. Table 2c: Risk of Bias Assessment for Quasi Experimental Studies. Table 2d: Risk of Bias Assessment for Cross Sectional Studies [file 13643_2022_1959_MOESM3_ESM.docx]

## Table 2a: Risk of Bias Assessment for Cohort Studies

|  | Q1 | Q2 | Q3 | Q4 | Q5 | Q6 | Q7 | Q8 | Q9 | Q10 | Q11 | Risk of Bias |
| --- | --- | --- | --- | --- | --- | --- | --- | --- | --- | --- | --- | --- |
| 20 |  |  |  |  |  |  |  |  |  |  |  |  |
| 27 |  |  |  |  |  |  |  |  |  |  |  |  |
| 97 |  |  |  |  |  |  |  |  |  |  |  |  |

## Table 2b: Risk of Bias Assessment for Randomized Trials

|  | Q1 | Q2 | Q3 | Q4 | Q5 | Q6 | Q7 | Q8 | Q9 | Q10 | Q11 | Q12 | Q13 | Risk of Bias |
| --- | --- | --- | --- | --- | --- | --- | --- | --- | --- | --- | --- | --- | --- | --- |
| 68 |  |  |  |  |  |  |  |  |  |  |  |  |  |  |

## Table 2c: Risk of Bias Assessment for Quasi Experimental Studies

|  | Q1 | Q2 | Q3 | Q4 | Q5 | Q6 | Q7 | Q8 | Q9 | Risk of Bias |
| --- | --- | --- | --- | --- | --- | --- | --- | --- | --- | --- |
| 71 |  |  |  |  |  |  |  |  |  |  |
| 53 |  |  |  |  |  |  |  |  |  |  |

## Table 2d: Risk of Bias Assessment for Cross Sectional Studies

|  | Q1 | Q2 | Q3 | Q4 | Q5 | Q6 | Q7 | Q8 | Risk of Bias |
| --- | --- | --- | --- | --- | --- | --- | --- | --- | --- |
| 16 |  |  |  |  |  |  |  |  |  |
| 17 |  |  |  |  |  |  |  |  |  |
| 21 |  |  |  |  |  |  |  |  |  |
| 22 |  |  |  |  |  |  |  |  |  |
| 24 |  |  |  |  |  |  |  |  |  |
| 28 |  |  |  |  |  |  |  |  |  |
| 29 |  |  |  |  |  |  |  |  |  |
| 33 |  |  |  |  |  |  |  |  |  |
| 34 |  |  |  |  |  |  |  |  |  |
| 35 |  |  |  |  |  |  |  |  |  |
| 36 |  |  |  |  |  |  |  |  |  |
| 50 |  |  |  |  |  |  |  |  |  |
| 51 |  |  |  |  |  |  |  |  |  |
| 63 |  |  |  |  |  |  |  |  |  |
| 66 |  |  |  |  |  |  |  |  |  |
| 75 |  |  |  |  |  |  |  |  |  |
| 77 |  |  |  |  |  |  |  |  |  |
| 92 |  |  |  |  |  |  |  |  |  |
| 93 |  |  |  |  |  |  |  |  |  |
| 98 |  |  |  |  |  |  |  |  |  |

## Legend

| Critical Appraisal Questions |
| --- |
| Yes |
| No |
| Unclear |
| Not applicable |

| Risk of Bias |
| --- |
| Low |
| Moderate |
| High |
